# Fatty Liver Disease in the Diabetic Population: A Cross-Sectional Study From Pakistan

**DOI:** 10.7759/cureus.85510

**Published:** 2025-06-07

**Authors:** Hassan Liaquat Memon, Shafaq Farooq, Muhammad Aslam, Ali Hyder, Khaild Tareen, Imran Ahmed, Raja Taha Yaseen Khan

**Affiliations:** 1 Gastroenterology, United Medical and Dental College, Creek General Hospital, Karachi, PAK; 2 Gastroenterology, Holy Family Hospital, Rawalpindi, PAK; 3 Gastroenterology, Madina Teaching Hospital, Faislabad, PAK; 4 Gastroenterology, Chandka Medical College, Shaheed Mohtarma Benazir Bhutto Medical University, Larkana, PAK; 5 Gastroenterology and Hepatology, Sheikh Khalifa Bin Zayed Al Nahyan Hospital, Quetta, PAK; 6 General Internal Medicine, King’s College Hospital, London, GBR; 7 Hepatogastroenterology, Sindh Institute of Urology and Transplantation, Karachi, PAK

**Keywords:** cardiovascular risk, dyslipidemia, frequency, non-alcoholic fatty liver disease, type 2 diabetes mellitus

## Abstract

Introduction

Non-alcoholic fatty liver disease (NAFLD) is a prevalent liver disorder, particularly among patients with type 2 diabetes mellitus (T2DM). The association between NAFLD and diabetes is bidirectional, with NAFLD increasing the risk of diabetes and vice versa. Therefore, this study aimed to determine the prevalence of NAFLD in diabetic individuals and evaluate the impact of metabolic and cardiovascular factors, along with the role of anti-diabetic medications in NAFLD progression.

Study methodology

After the approval from the United Medical and Dental College - Institutional Review Board (UMDC-IRB) (UMDC-IRB-2022/A-1713), this cross-sectional study was carried out at the Department of Gastroenterology, United Medical and Dental College from January 2023 to December 2023. A total of 250 diabetic patients aged between 30 and 65 years were enrolled in the study. Patients with a history of alcohol consumption, viral hepatitis, autoimmune hepatitis, and chronic kidney disease (CKD) stage 4 or higher were excluded from the study. Data collection involved demographic profiling, laboratory investigations (HbA1c, lipid profile, and liver function tests), and imaging assessment using an abdominal ultrasound to determine the presence or absence of fatty liver. Statistical analysis was conducted using SPSS version 25.0 (IBM SPSS Statistics for Windows, IBM Corp., Armonk, NY), with p ≤ 0.05 considered statistically significant.

Results

Among the 250 diabetic patients, 178 (71.2%) were diagnosed with NAFLD. Higher BMI (p ≤ 0.001), poor glycemic control (HbA1c > 8.5%) (p ≤ 0.001), and dyslipidemia (p ≤ 0.001) were significantly associated with the presence of NAFLD in the diabetic population. Hypertension (p ≤ 0.001) and coronary artery disease (p = 0.031) were also the factors more prevalent in the NAFLD group. Patients on glucagon-like peptide 1 (GLP-1) receptor agonists (p = 0.018) and sodium-glucose cotransporter-2 (SGLT2) inhibitors (p = 0.042) had a significantly lower prevalence of NAFLD.

Conclusion

NAFLD is highly prevalent among diabetic individuals, with significant cardiovascular implications. Routine screening and targeted interventions are essential to mitigate liver-related complications in this high-risk population.

## Introduction

Non-alcoholic fatty liver disease (NAFLD) has emerged as the most common liver disorder worldwide, particularly in individuals with metabolic conditions such as diabetes mellitus (DM) [1]. NAFLD is a broad-spectrum disease, ranging from simple hepatic steatosis (fat accumulation in the liver) to non-alcoholic steatohepatitis (NASH), fibrosis, cirrhosis, and hepatocellular carcinoma (HCC) [2]. The rising incidence of obesity, insulin resistance, and metabolic syndrome has fueled the prevalence of NAFLD, making it a significant global health concern [1].

Diabetes, particularly type 2 diabetes mellitus (T2DM), plays a critical role in the pathogenesis and progression of NAFLD. The relationship between these conditions is bidirectional: NAFLD increases the risk of developing diabetes, while diabetes exacerbates liver disease progression [3]. Studies have shown that approximately 70% of patients with T2DM have some degree of NAFLD, and they are at a higher risk of developing advanced liver disease, including cirrhosis and liver-related mortality [4-6]. Moreover, NAFLD in diabetic patients is associated with an increased risk of cardiovascular disease (CVD), chronic kidney disease (CKD), and overall mortality [7].

The pathophysiological mechanisms linking NAFLD and diabetes include insulin resistance, lipotoxicity, oxidative stress, chronic inflammation, and genetic predisposition. Insulin resistance is a central driver in both conditions, leading to increased hepatic fat accumulation, inflammation, and fibrosis [8]. Additionally, adipose tissue dysfunction, altered lipid metabolism, and gut microbiota dysbiosis contribute to disease progression [9].

The management of NAFLD in diabetes is complex and requires a multi-faceted approach. Lifestyle modifications, including weight loss, dietary changes, and physical activity, are the cornerstone of treatment [10]. Pharmacological interventions, such as glucagon-like peptide 1 (GLP-1) receptor agonists, sodium-glucose cotransporter-2 (SGLT2) inhibitors, and pioglitazone, have shown promise in improving both hepatic and metabolic outcomes [11]. However, there is still no FDA-approved medication specifically for NAFLD, underscoring the need for further research and novel therapeutic strategies.

The increasing prevalence of diabetes worldwide has made NAFLD a significant public health concern, particularly in diabetic populations where it exacerbates liver-related morbidity and increases cardiovascular risk [7]. In Pakistan, multiple studies have been done previously evaluating the frequency and non-invasive parameters suggestive of NAFLD. Previously, Khan et al. determined the frequency of fatty liver disease in lean patients [5]. However, the data are limited regarding the research on the association between diabetes and NAFLD. Shaikh et al. found the prevalence of diabetes to be about 26% in the lean Pakistani population with NAFLD [3].

Therefore, the main aim of this study was not only to identify the frequency of NAFLD in the diabetic population but also to compare the impact of cardiovascular and antidiabetic medications on the presence or absence of NAFLD in the diabetic population.

## Materials and methods

Study design and study place

After the approval from United Medical and Dental College - Institutional Review Board (UMDC-IRB), this cross-sectional study was carried out at the department of gastroenterology, UMDC, Korangi Creek Hospital, Karachi, from 1st January 2023 to 31st December 2023. All the patients aged between 30 and 65 years diagnosed with T2DM were enrolled in the study. While patients with a history of viral hepatitis (HBV, HCV), alcoholic liver disease, autoimmune liver diseases, or those with CKD (stage 4 or higher), patients on medications known to cause hepatic steatosis (e.g., corticosteroids, amiodarone), pregnant, or lactating women were excluded from the study.

Patients were enrolled using the technique of non-probability consecutive sampling. Based on previous estimates, NAFLD was observed in about 70% of patients with diabetes. Taking a margin of error of 7.5% and a confidence level of 95%, a total of 250 patients were needed for this study.

Data collection procedure

After the enrollment in the study, participants underwent a comprehensive clinical assessment, including demographic data collection (age, gender, BMI, medical history), laboratory investigations, including liver function tests (alanine aminotransferase (ALT), aspartate aminotransferase (AST), and gamma-glutamyl transferase (GGT)), lipid profile, fasting blood glucose, and HbA1c. Ultrasound of the abdomen was performed in each patient for the presence or absence of fatty liver disease (hyperechoic liver).

Data analysis

Data was analyzed using SPSS software version 25.0 (IBM SPSS Statistics for Windows, IBM Corp., Armonk, NY). Continuous variables were expressed as mean ± standard deviation, while categorical variables were presented as frequencies and percentages. Statistical tests, such as chi-square tests and Student t-test, were used for the comparison of categorical and continuous variables, respectively. A p-value of <0.05 was considered statistically significant.

## Results

A total of 250 diabetic patients were included in this study. The gender distribution showed a higher male prevalence, with 140 (56%) males and 110 (44%) females. The mean age of the study population was 52.6 ± 8.7 years. At the time of presentation, hypertension and coronary artery disease (CAD) were observed in 146 (58.4%) and 60 (24%) patients, respectively. Of 250 patients, 96 (38.4%) and 100 (40%) patients were overweight (25-29.9 kg/m²) and obese (≥30 kg/m²), respectively. HbA1c ≥ 7% was observed in 167 (66.8%) patients at the time of presentation. Ultrasound abdomen showed fatty liver in 178 (71.2%) (Table 1).

**Table 1:** Baseline characteristics of the diabetic population (n = 250) ALT: alanine transaminase; AST: aspartate transaminase; BMI: body mass index; HbA1c: hemoglobin A1c; HDL: high density lipoprotein; LDL: low density lipoprotein Reference ranges: Hb: 12-16 (g/dL); total bilirubin: 0.8-1.0 (mg/dL); AST: up to 40 (U/L); ALT: up to 40 (U/L); triglycerides: up to 150 (mg/dL); HDL: 40-60 (mg/dL); LDL: up to 120 (mg/dL)

Variable		n (%)
Mean age (years)		52.6 ± 8.7
Gender	Males	140 (56)
	Females	110 (44)
BMI distribution	Normal weight (<25 kg/m²)	54 (21.6)
	Overweight (25-29.9 kg/m²)	96 (38.4)
	Obese (≥30 kg/m²)	100 (40)
Fatty liver	Yes	178 (71.2)
	No	72 (28.8)
HbA1c levels	<7%	83 (33.2)
	≥7%	167 (66.8)
Cardiovascular complications	Hypertension	146 (58.4)
	Coronary artery disease	60 (24)
Hemoglobin (g/dL)		13.2 ± 1.8
Bilirubin (mg/dL)		0.9 ± 0.3
ALT (U/L)		52.3 ± 10.2
AST (U/L)		44.5 ± 9.8
Triglycerides (mg/dL)		210 ± 45
HDL (mg/dL)		38 ± 7
LDL (mg/dL)		128 ± 28

Anthropometric and glycemic parameters

BMI was significantly associated with NAFLD, with a higher prevalence of NAFLD among overweight and obese individuals (p ≤ 0.001). Among those diagnosed with NAFLD, 96 (54%) patients were obese (BMI ≥ 30 kg/m²), 64 (36%) patients were overweight (BMI 25-29.9 kg/m²), and only 18 (10%) patients had normal BMI (<25 kg/m²). In contrast, a higher proportion of non-NAFLD individuals, i.e., 36 (50%), had normal BMI.

HbA1c levels were significantly higher in the NAFLD group, with 160 (90%) of NAFLD patients having HbA1c > 7%, compared to only seven (10%) in the non-NAFLD group (p ≤ 0.001) (Table 2).

**Table 2:** Comparison of baseline characteristics, cardiovascular complications, and effect of anti-diabetic medications in terms of presence or absence of NAFLD in diabetic population (n = 250) *The chi-square test is used for the calculation of p-values for categorical variables. **Student t-test is used for the calculation of p-values of continuous variables. ALT: alanine transaminase; AST: aspartate transaminase; BMI: body mass index; HbA1c: hemoglobin A1c; HDL: high density lipoprotein; LDL: low density lipoprotein Reference ranges: AST: up to 40 (U/L); ALT: up to 40 (U/L); triglycerides: up to 150 (mg/dL); HDL: 40-60 (mg/dL); LDL: up to 120 (mg/dL)

Variable		NAFLD present (n = 178) n (%)	NAFLD absent (n = 72) n (%)	Chi-square value*/t-test value**	p-value
Gender	Males	100 (56.2)	40 (55.6)	0.02	0.08
	Females	78 (43.8)	32 (44.4)		
BMI distribution	Normal weight (<25 kg/m²)	18 (10)	36 (50)	48.5	≤0.001
	Overweight (25-29.9 kg/m²)	64 (36)	32 (44.4)		0.012
	Obese (≥30 kg/m²)	96 (54)	4 (5.6)		≤0.001
HbA1c levels	<7%	18 (10)	65 (90.3)	32.6	≤0.001
	≥7%	160 (90)	7 (9.7)		≤0.001
Hypertension	Yes	119 (66.8)	27 (37.5)	16.9	≤0.001
	No	59 (33.2)	45 (62.5)		
Coronary artery disease (CAD)	Yes	50 (28)	10 (14)	4.9	0.031
	No	128 (72)	62 (86)		
Anti-diabetic treatment	GLP-1 receptor agonists	45 (25.2)	22 (30.6)	5.7	0.018
	SGLT2 inhibitors	36 (20.2)	25 (34.7)	5.2	0.042
	Pioglitazone	27 (15.2)	7 (9.7)	0.8	0.147
	Insulin therapy	70 (39.4)	18 (25)	4.3	<0.01
Mean age (years)		54.2 ± 8.5	52.1 ± 7.9	3.1	0.063
ALT (U/L)		60.5 ± 11.8	38.4 ± 8.2	13.4	≤0.001
AST (U/L)		50.2 ± 9.5	32.8 ± 6.5	14.9	≤0.001
LDL (mg/dL)		135 ± 30	115 ± 26	5.2	≤0.001
Triglycerides (mg/dL)		230 ± 48	170 ± 38	11.2	≤0.001
HDL (mg/dL)		35 ± 6	45 ± 8	-12.04	0.021

Liver function and lipid profile

Liver enzyme levels were markedly elevated in patients with NAFLD. The mean ALT level was significantly higher in the NAFLD group (60.5 ± 11.8 U/L) compared to non-NAFLD individuals (38.4 ± 8.2 U/L) (p ≤ 0.001). A similar trend was observed in AST levels, with NAFLD patients having a mean AST of 50.2 ± 9.5 U/L, significantly higher than 32.8 ± 6.5 U/L in the non-NAFLD group (p ≤ 0.001) (Table 2).

Dyslipidemia was more prevalent among NAFLD patients, with higher triglyceride levels (230 ± 48 mg/dL vs. 170 ± 38 mg/dL) (p ≤ 0.001) and higher LDL levels (135 ± 30 mg/dL vs. 115 ± 26 mg/dL) (p ≤ 0.001). Conversely, HDL levels were significantly lower in NAFLD patients (35 ± 6 mg/dL vs. 45 ± 8 mg/dL) (p = 0.021), reinforcing the strong association between NAFLD and metabolic syndrome (Table 2).

Cardiovascular complications

Cardiovascular disorders were more prevalent among NAFLD patients. Hypertension was significantly higher in the NAFLD group (119 (66.8%)) compared to the non-NAFLD group (27 (37.5%)) (p ≤ 0.001). Additionally, CAD was also significantly more common in NAFLD patients (p = 0.031) (Table 2).

Impact of anti-diabetic medications

The choice of anti-diabetic medications appeared to influence the presence of NAFLD. Patients on GLP-1 receptor agonists (e.g., liraglutide, semaglutide) had a lower prevalence of NAFLD (25% in the NAFLD group vs. 30.6% in the non-NAFLD group) (p = 0.018). Similarly, 20% of NAFLD patients were on SGLT2 inhibitors compared to 35% in the non-NAFLD group (p = 0.042), suggesting a protective effect of these agents. On the contrary, patients on insulin therapy for diabetes had a higher prevalence of NAFLD (40% in the NAFLD group vs. 25% in the non-NAFLD group) (p ≤ 0.001) (Table 2).

## Discussion

The findings of this study showed a high prevalence of NAFLD in diabetic patients, in agreement with previous studies highlighting the strong association between NAFLD and diabetes. In this study, we found 71.2% of diabetic patients had NAFLD, which was similar to the findings observed in previous studies showing up to 70% of T2DM patients having NAFLD [1,2]. This confirms the evidence that NAFLD is extremely common in diabetic patients and, therefore, an essential comorbidity needing early identification and management.

The prevalence in this study (71.2%) closely approximates results from previous studies. Younossi et al., in a meta-analysis in 2019, estimated 55-75% of T2DM patients to have NAFLD and confirmed our findings [12]. Also, a Pakistani study by Shaikh et al. revealed a prevalence of diabetes in 26% of lean NAFLD patients, indicating that diabetes is an important risk factor for fatty liver in non-obese individuals as well [3]. Our study confirms this by proving NAFLD to be extremely common in overweight and lean diabetic individuals as well, emphasizing the metabolic subtlety of fatty liver disease.

Our study showed a strong relationship between BMI and NAFLD, with the highest prevalence in obese patients (54%). This concurs with the literature internationally identifying obesity as a significant risk factor for NAFLD in diabetics. Previous studies have shown that BMI is a strong predictor of hepatic steatosis in diabetics and have also emphasized the role of metabolic impairment [13,14]. In addition, the HbA1c level in the NAFLD group was significantly higher in 90% of NAFLD patients with HbA1c ≥7%, compared to only 10% in the non-NAFLD group (p ≤ 0.001). These findings are in agreement with studies where poor glycemic control leads to accumulation of hepatic fat and inflammation and an increased risk of fibrosis in the liver [15].

The liver enzyme levels (ALT and AST) were significantly elevated in NAFLD patients in our study, with mean values of 60.5 ± 11.8 U/L and 50.2 ± 9.5 U/L for ALT and AST, respectively. These findings are similar to those observed in the previous studies, where it was established that elevated liver enzymes are a feature of NAFLD in diabetics [16]. Dyslipidemia was also present in increased frequencies in NAFLD patients with significantly elevated levels of triglyceride and LDL and decreased levels of HDL. This result affirms studies where it has been established that dyslipidemia plays a significant role in the etiology of NAFLD in diabetes by inducing increased fat accumulation in the liver and oxidative stress [17].

In our study, hypertension and CAD were far more common in NAFLD patients, with 66.8% of NAFLD patients having hypertension as compared to 37.5% in the non-NAFLD group (p ≤ 0.001). CAD was also far greater in NAFLD patients (28% compared to 14%, p = 0.031). These findings are in agreement with previous studies pointing toward NAFLD as an independent risk factor for CVD in diabetic patients. In a study by Targher et al. (2005), diabetic patients with NAFLD had two to three times higher risk of cardiovascular events compared to those with no NAFLD [18].

The prevalence of NAFLD in relation to anti-diabetic therapy was also evaluated. Our study showed a reduced prevalence of NAFLD in patients treated with GLP-1 receptor agonists and SGLT2 inhibitors, suggesting a potential protective effect. Specifically, 25.2% of NAFLD patients were treated with GLP-1 receptor agonists compared to 30.6% in the non-NAFLD group (p = 0.018). Similarly, 20.2% of NAFLD patients were treated with SGLT2 inhibitors compared to 34.7% in the non-NAFLD group (p = 0.042). These findings are in agreement with reports suggesting GLP-1 receptor agonists and SGLT2 inhibitors reduce hepatic steatosis by attenuating insulin resistance and hepatic fat accumulation [19]. In contrast, insulin-treated patients had a higher prevalence of NAFLD (39.4% vs. 25%, p < 0.001), in agreement with previous evidence suggesting exogenous insulin leads to hepatic fat accumulation due to its anabolic effect on lipid metabolism [20].

Despite its strengths, this study has several limitations. First, the cross-sectional design of the study limits the ability to establish causality between diabetes and NAFLD progression, making it difficult to determine temporal relationships. Second, the study was conducted at a single center in Pakistan, which may limit its generalizability to diverse populations with different genetic, environmental, and dietary factors, and therefore, we recommend the need for multi-center studies. Third, the lack of longitudinal data prevents an evaluation of disease progression and the long-term effects of anti-diabetic medications on NAFLD, underscoring the need for future prospective studies to validate these findings, as these factors could influence the effects of the drugs and the prevalence of NAFLD. Fourth, there are certain limitations of ultrasound, such as lower sensitivity and higher potential for missing mild fatty liver; therefore, we suggest that more sensitive diagnostic methods (e.g., magnetic resonance imaging-derived proton density fat fraction (MRI-PDFF), elastography, liver biopsy) are desirable for future research.

## Conclusions

The results of this study not only verify the high prevalence of NAFLD in diabetic patients but also highlight the association of high BMI and erratic blood sugar levels with the presence of NAFLD in the diabetic population, with a significant risk of cardiovascular events if not addressed promptly. Our results also suggested the possible role of anti-diabetic medications like GLP-1 receptor agonists and SGLT2 inhibitors in reducing hepatic fat accumulation. However, longitudinal and intervention studies are needed to determine the long-term benefits of such therapy in the management of NAFLD. Considering the significant prevalence of NAFLD in diabetics, screening for fatty liver disease on a regular basis should be made an essential part of the management of diabetes. Future research should aim at investigating novel treatments to reduce the development of NAFLD, particularly in the diabetic population.
